# Anodal tDCS Over the Left DLPFC Did Not Affect the Encoding and Retrieval of Verbal Declarative Information

**DOI:** 10.3389/fnins.2017.00452

**Published:** 2017-08-08

**Authors:** Gabriel A. de Lara, Philipp N. Knechtges, Walter Paulus, Andrea Antal

**Affiliations:** Department of Clinical Neurophysiology, University Medical Center Goettingen, Georg-August University of Goettingen Göttingen, Germany

**Keywords:** tDCS, verbal associative learning, verbal long-term memory, DLPFC

## Abstract

Several studies imply that anodal transcranial direct current stimulation (tDCS) over the left dorsolateral prefrontal cortex (DLPFC) can modulate the formation of verbal episodic memories. The aim of this study was to test if tDCS through a multi-electrode Laplacian montage over the left DLPFC could differentially modulate declarative memory performance depending on the application phase. Two groups of healthy participants (*n* = 2 × 15) received 1 mA anodal or sham stimulation for 20 min during the encoding or during the recall phase on a delayed cued-recall, using a randomized, double-blinded, repeated-measures experimental design. Memory performance was assessed at two time points: 10 min and 24 h after learning. We found no significant difference between anodal and sham stimulation with regard to the memory scores between conditions (stimulation during encoding or recall) or between time points, suggesting that anodal tDCS over the left DLPFC with these stimulation parameters had no effect on the encoding and the consolidation of associative verbal content.

## Introduction

Low-intensity transcranial electrical brain stimulation (TES) has the potential to further improve our knowledge about the functional and neural correlates of declarative memory, by directly manipulating the neural activity of targeted brain areas before or during the performance of a given task. Previous studies in this research field have found promising improvements in subjects' recognition of encoded material when transcranial direct current (tDCS), alternating current (tACS), or oscillatory tDCS was applied in either the learning and/or in the recognition phase (Marshall et al., 2006; Jacobson et al., 2012; Javadi et al., 2012; Javadi and Walsh, 2012; Ambrus et al., 2015; Pisoni et al., 2015). Among the above-mentioned techniques, tDCS is one of the most extensively used TES methods. It is thought that tDCS is capable of inducing polarity-dependent, relatively long-lasting changes in the human brain, probably either by de- or hyperpolarising neurons' resting membrane potentials and causing a reversible change in the balance of excitatory-inhibitory cortical activity (for recent reviews see Hartwigsen et al., 2015; Woods et al., 2016; Fertonani and Miniussi, 2017).

A meta-analysis of fMRI studies on episodic memory showed left lateralized effects for the encoding of verbal material, arguing in favor of the involvement of the prefrontal cortex (Kim, 2011). Additionally, results from non-invasive brain stimulation studies suggest that the left dorsolateral prefrontal cortex (DLPFC) may be involved in both the encoding and retrieval of verbal content (Manenti et al., 2012). Furthermore, several sources of recent experimental data indicate that the application of anodal tDCS over the left DLPFC during learning results in improvements in different cognitive tasks, including the encoding of semantic material (e.g., Brunoni and Vanderhasselt, 2014; Dedoncker et al., 2016b; Kim et al., 2016; Hill et al., 2017), although conflicting results were also reported (e.g., Tremblay et al., 2014). Further research considered that the stimulation timing might be critical (Dedoncker et al., 2016a, b; e.g., before or during the performance of the task), with the results usually showing a small, but significant, effect on accuracy and reaction time in working memory, when tested after the application of anodal tDCS.

A recent study tested the hypothesis that long-term associative-memory engrams are stored in an excitatory-inhibitory balance in neuronal ensembles. Learning is assumed to change synaptic strength, which is disrupted during this process, with the new excitatory connections being rebalanced afterwards by inhibitory GABAergic mechanisms (Barron et al., 2016). They showed that by unmasking inhibitory connections using anodal tDCS to downregulate cortical GABA concentration after learning, significant improvement could be obtained in associative memory, which correlated with a decreased GABA level in the targeted area.

To clarify whether anodal tDCS directed to the left prefrontal cortex could indeed significantly modulate the encoding or retrieval of verbal associative learning, we chose to apply 20 min of tDCS, as it constitutes common standard in the field for cognitive paradigms (Hill et al., 2017). For this we designed two experiments with different stimulation time points: in the first group, anodal tDCS was applied before and during learning, in order to augment learning-induced neuronal plasticity. In the second group, stimulation was administered before and during the recall phase 24 h after learning with the aim to rebalance inhibitory plasticity after learning, as previous studies showed that anodal tDCS can effectively decrease the GABA level (Stagg, 2014; Stagg et al., 2014; Barron et al., 2016). While after learning (and during “forgetting”) the new excitatory connections are frequently rebalanced by inhibition, we hypothesized that the stimulation during the recall phase might induce enhanced memory performance by downregulating the increased GABA level, compared to sham stimulation, and similarly, during the encoding phase.

## Materials and methods

### Participants

Thirty healthy, young adult, right-handed, native German speakers with normal or corrected-to-normal vision were recruited, after giving their informed consent. They were assigned to two groups of 15 participants each (group 1: eight females, mean age 24.8 ± 3.5, age range 18–30; group 2: seven females, mean age 24.6 ± 3, age range 18–31). They had no history of neuropsychiatric or brain disorders. The participants were naïve to the applied task and were reimbursed for their participation. The project was approved by the ethics committee of the University Medical Center Göttingen and was conducted accordingly to the Declaration of Helsinki. No participant reported adverse effects.

### Experimental procedure

**Figure 1:**
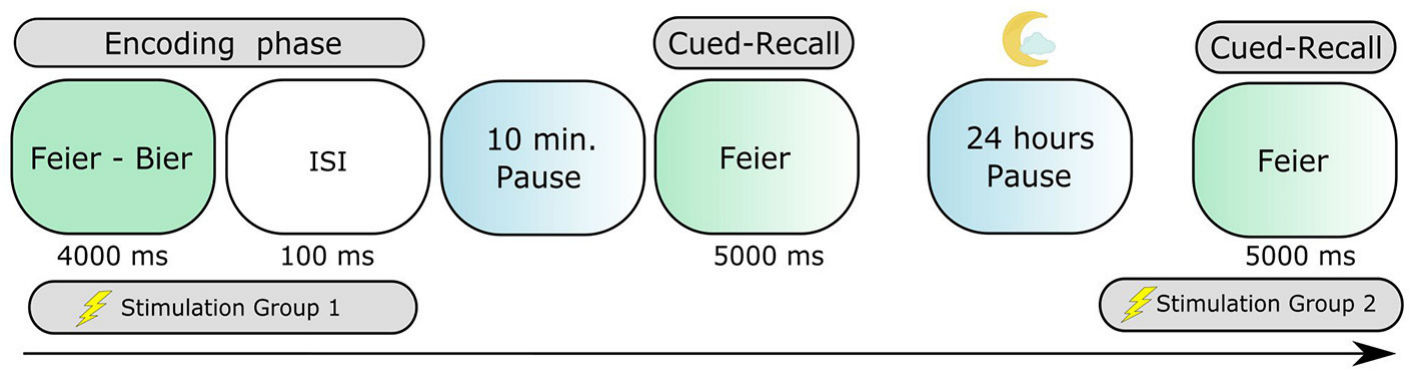
The paired-associate learning task assessing episodic long-term memory. Participants learned 52 semantically-related German paired nouns. The cued-recall testing consisted in verbally expressing the second word of the pair, always 10 min and again 24 h after the encoding phase.

A randomized, double-blind, repeated-measures, placebo-controlled design was used, with each participant taking part only in one experimental group. Every participant underwent two stimulation conditions (anodal tDCS and sham) with blocks of two experiments related to one condition (stimulation during encoding—group 1 or recall—group 2) separated by 24 h, generating a total of four sessions (Figure 1). The first and the third sessions consisted of a (1) learning phase, combined with the application of tDCS in the group 1, (2) a 10-min pause, and (3) a first cued-recall. The second and fourth sessions were composed of a second cued-recall to assess memory overnight consolidation, combined with tDCS only in group 2. Each stimulation session was separated by at least a 5-day interval to avoid carryover effects. To minimize the well-known learning effect in word-list memory tasks, the order of real and placebo conditions were counterbalanced across participants. At the beginning of the first session, the subjects received written instruction about the task and were informed about the experimental procedures. The participants also filled in an additional indicators questionnaire and were debriefed after the stimulation sessions.

### Stimulation protocol

tDCS was delivered by using a certified NeuroConn Multichannel stimulator (Ilmenau, Germany). We used a set of five 3 cm^2^ rubber-round electrodes with Ten20 paste as conductivity mean. Both of the groups received 1.0 mA of tDCS applied for 20 min continuously during the learning phase of the task (group 1) or during the second day's cued-recall (group 2). For group 1, the stimulation was started 12 min before presentation of the learning material, and then continued during it (learning duration was 8 min); for group 2, the stimulation started 15 min before and then continued during the cued-recall (which lasted 5 min). During the real (anodal tDCS) and sham stimulation the current was ramped up for 10 s in the beginning until reaching the programmed intensity, and then ramped down for 10 s at the end. In the sham condition, the current was additionally applied for 30 s and then discontinued. The impedances were kept below the limit of 5 kΩ as measured by the device.

### Montage

**Figure 2:**
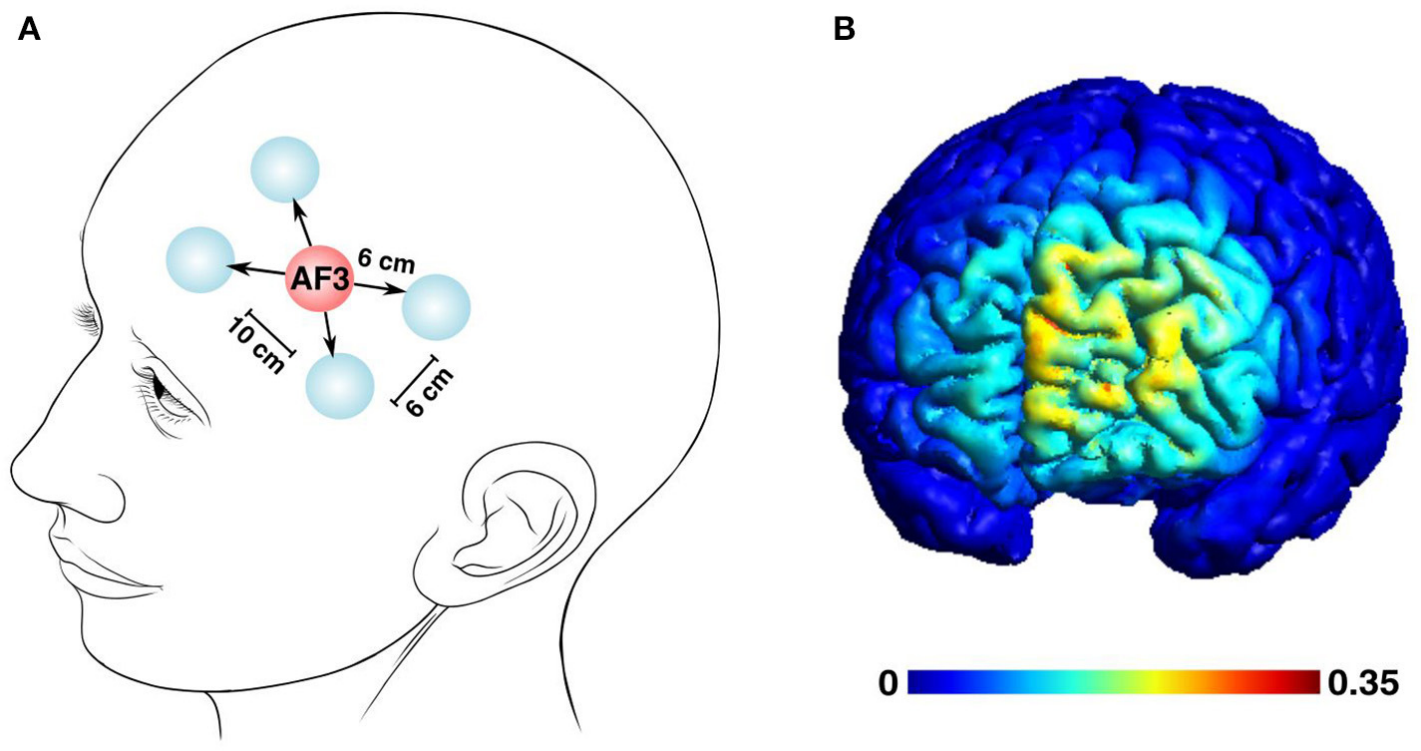
The multi-electrode tDCS left pre-frontal montage and the estimated distribution of the tDCS-generated electric field. **(A)** A five-electrode Laplacian montage to deliver the current was centered over the AF3 position, surrounded by 4 return electrodes. The distances between the electrodes were set as follows: central and return electrodes, 6 cm; adjacent return electrodes, 6 cm; distance between the medial and lateral return electrodes, 10 cm (Human head modified from Patrick J. Lynch's illustration, distributed under a CC-BY 2.5 license.) **(B)** The estimated electric field distribution is color-coded to the intensity scale, with the maximum field strength reaching 0.35 mV/mm.

The positioning of the electrodes was standardized and kept constant across the experiments as suggested by modeling studies (Saturnino et al., 2015), with the plugs and cables always turned in a medial-to-lateral direction. The Laplacian multi-electrode montage, designed to answer our research hypothesis, was composed of a central anodal electrode over the AF3 position (according to the international 10–20 EEG system) and four surrounding return electrodes with 6 cm distance from the central one, and 10 cm distance between the medial and lateral electrodes (Figure 2A). A realistic finite-element model (Figure 2B) to evaluate the extension and precision of our anatomical target and to estimate the distribution of the electric field was generated in SIMNIBS 2.0.1 (Thielscher et al., 2015). The model accounts for white matter anisotropy and the following conductivity for these anatomical components: scalp (σ = 0.465 S/m), bone (σ = 0.010 S/m), cerebrospinal fluid (σ = 1.654 S/m), gray matter (σ = 0.275 S/m), and white matter (σ = 0.126 S/m). The tetrahedral volume mesh post-processing and visualization was generated through Gmsh (Geuzaine and Remacle, 2009).

### Task

A verbal-associative learning task (Figure 1), shown in previous studies to be sensitive in the capture of effects of non-invasive brain stimulation in declarative memory (Marshall et al., 2006; Garside et al., 2015), was utilized in order to assess verbal episodic memory. In this paradigm, the participants were asked to memorize semantically related word-pairs presented one at a time. For each experimental condition, a different list with a total of 54 word-pairs composed of associated German nouns was presented on a monitor, where 8 (4 in the beginning and 4 in the end) of them were dummy pairs to buffer recency and primacy effects. The order of the lists was randomized across subjects and conditions. Each correct answer was granted two points, with one point given to late or partially correct (morphologically incorrect) answers, totaling 92 possible points (100% performance) to score. The dummy pairs were excluded from the data analysis. The subjects were exposed to each word-pair for 4 s with an inter-stimulus interval of 100 ms, thereby learning the list two times in a different, randomized order. With two different time delays (10 min and 24 h), the participants' memory performance was subsequently tested with a cued-recall in a forward-recall manner, where each stimuli was presented for 5 s. The stimuli in the two cued-recalls were presented in two different, randomized orders. During the 10-min pause following the learning phase, the participants stayed seated and had no other activity or verbal interaction with the researcher. No feedback was given about the correctness of the answers. The task was conducted using Presentation software (Neurobehavioral Systems Inc., Albany, CA, USA).

### Statistical analysis

**Figure 3:**
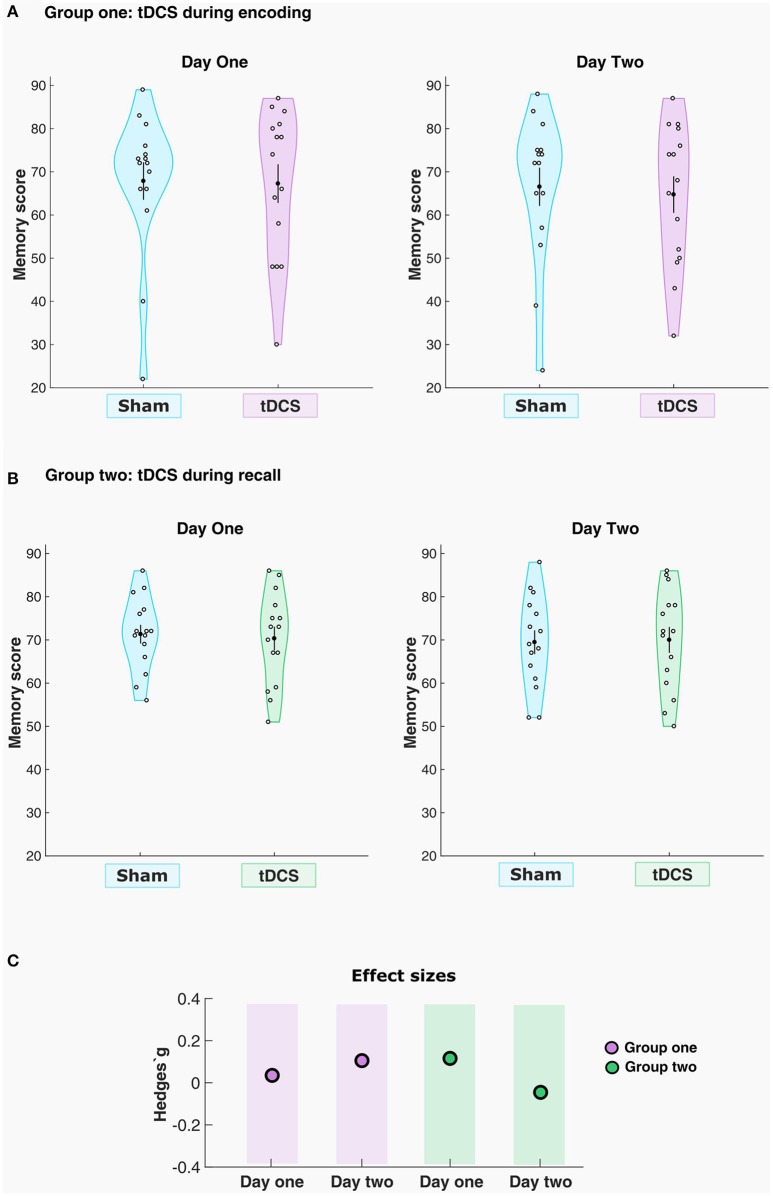
tDCS had no significant effects on memory performance. The violin plots indicate the density of the sample distribution across the *y*-values. Mean and standard error of the mean (SEM) are shown in each plot as the black dot and the black line **(A)** Memory score for sham and real stimulation conditions for each participant in group one, day 1 and 2, respectively. **(B)** Memory score for sham and real stimulation conditions for each participant in group two, day 1 and 2, respectively. **(C)** Effect sizes for the real tACS conditions across the two groups.

In a first step, all the groups underwent null hypothesis significance testing to compare their behavioral performance in the task. As in the first experimental group two variables were non-normally distributed, a related-samples Wilcoxon signed rank test was employed in order to compare memory performance between sham and real tDCS, both for the first and second testing days. To rule out baseline differences that could impact the outcomes in our parallel-group design, a non-parametric independent-samples Mann–Whitney *U*-test was used to compare the sham condition performances on both days. In addition to the null hypothesis significance testing, we ran Bayesian analyses to verify the amount of evidence for the null or the alternative hypothesis given in our dataset (Rouder et al., 2009). One-sided JSZ Bayes Factors (BF_01_) were computed in JASP (version 0.8.1.2) to estimate how likely the null hypothesis (there are no differences between the conditions) could be observed under the alternative hypothesis (there are differences), with a Cauchy prior width of 0.707. We also calculated the effect size for all the real conditions compared with sham for the respective groups (Figure 3C). The calculations were performed with the Measures of Effect Size toolbox for MATLAB, which provides a corrected and unbiased *Hedges'g* estimation for small paired samples (Hentschke and Stuttgen, 2011).

### Arousal levels and sleep quality indicators

To control for two variables that directly influence memory encoding and retrieval (Diekelmann and Born, 2010; Rutishauser et al., 2010), we asked the participants to report their arousal levels and sleep time and quality in the previous night (Table 1). The arousal was assessed on a self-report scale from 1 to 10 (1 = very tired, 10 = totally awake). Sleep quality was measured through self-report, including the number of hours subjects slept during the previous night (Likert scale, 1–5 points continuum; 1 = very bad, 5 = very good). All the indicators were analyzed using the non-parametric paired samples Wilcoxon signed rank test.

**Table 1:** Results for the sleep and arousal indicators.

	**Condition**	**Value**	**Test**
**SLEEP**			
**Group 1**			
Amount of hours	Session 1		*p* = 0.092
	Real	7.5 ± 0.81	
	Sham	7.1 ± 1.1	
	Session 2		*p* = 0.223
	Real	7.2 ± 0.9	
	Sham	6.7 ± 1.5	
Sleep quality	Session 1		*p* = 0.683
	Real	3.4 ± 1.0	
	Sham	3.6 ± 1.0	
	Session 2		*p* = 0.666
	Real	3.9 ± 0.8	
	Sham	3.9 ± 1.0	
**Group 2**			
Amount of hours	Session 1		*p* = 0.138
	Real	7.5 ± 0.9	
	Sham	6.9 ± 1.2	
	Session 2		*p* = 0.634
	Real	7.2 ± 1.1	
	Sham	7.3 ± 1.2	
Sleep quality	Session 1		*p* = 0.490
	Real	3.6 ± 0.7	
	Sham	3.4 ± 0.9	
	Session 2		*p* = 0.008
	Real	3.4 ± 0.9	
	Sham	4.3 ± 0.6	
**AROUSAL**			
**Group 1**			
First cued recall	Real	7.1 ± 1.2	*p* = 0.888
	Sham	7.2 ± 1.4	
Second cued recall	Real	7.4 ± 0.9	*p* = 0.392
	Sham	7.6 ± 1.3	
**Group 2**			
First cued recall	Real	7.9 ± 1.1	*p* = 0.006
	Sham	6.3 ± 1.8	
Second cued recall	Real	7.0 ± 1.4	*p* = 0.848
	Sham	7.0 ± 1.9	

## Results

### Memory accuracy

The results with regard to memory performance in the paired-associative learning task are summarized in Figure 3. The task permitted an absolute maximum numerical score of 92 points, and the results are plotted in original values for all days when memory accuracy was measured in a cued-recall fashion. In the first group (*n* = 15), where the participants received the stimulation during encoding, a small numerical difference in memory performance was observed between the real (67.2 ± 17.3) condition compared to sham (67.8 ± 16.8) stimulation in the first recall test, and also between sham (66.5 ± 17.1) and real tDCS (64.7 ± 16.4) on the second day of recall. The related-samples Wilcoxon signed rank test revealed no statistically significant effect of the stimulation condition, for either the first day (*Z* = −0.057, *p* = 0.955, Hedges'g = 0.03) or the second day (*Z* = 0.664, *p* = 0.506, Hedges'g = 0.10) of testing. The computed Bayes Factor showed moderate evidence in favor of the null hypothesis on the first cued-recall (BF_01_ = 3.719), where the null hypothesis is 3.719 times more likely to be observed that the alternative hypothesis given this dataset. For the second cued-recall, Bayes Factor also showed moderate evidence for the null hypothesis (BF_01_ = 3.237), i.e., it is 3.237 times more likely to be observed than the alternative given the present data.

For the second group (*n* = 15), where the participants received the tDCS during retrieval, the first cued-recall showed a slight numerical difference in memory performance between the anodal tDCS group (70.3 ± 10.6) compared to sham (71.4 ± 8.3). On the delayed cued-recall, memory performance was also slightly different between the real stimulation (70.0 ± 11.6) compared to sham (69.4 ± 10.6). The related samples Wilcoxon signed rank test revealed no statistically significant differences in memory performance between sham and real tDCS for either the first (*Z* = −0.711, *p* = 0.477, Hedges'g = 0.11) or the second cued-recall (*Z* = −0.566, *p* = 0.572, Hedges'g = −0.04). Here, Bayes Factor showed moderate evidence in favor of the null hypothesis (BF_01_ = 3.326) for the first cued-recall, where the null is 3.326 times more likely to be observed than the alternative. For the second cued-recall, Bayes Factor also showed moderate evidence for the null hypothesis (BF_01_ = 3.737), being 3.737 times more likely to be observed given the actual data.

Moreover, the independent samples Mann–Whitney *U*-test revealed no significant difference in sham performance between the groups, for neither the first cued-recall (*U* = 112.500, *p* = 1.000), or for the second cued-recall (*U* = 119.500, *p* = 0.771).

### Sleep quality and arousal indicators

A summary of the sleep quality and arousal data collected on the day of the experiments is reported in Table 1. In the first group (stimulation during encoding), the number of hours slept the night before the experimental session showed no significant difference between real and sham condition for session one or session two. There was also no significant difference in sleep quality report on the night before the experiments between real and sham for either of the sessions. Similarly, Wilcoxon signed rank test showed no significant difference in the arousal levels between sham and real stimulation for the first and second day of memory cued-recall testing.

For the second group (stimulation during retrieval), the amount of reported hours slept on the night before the experiments showed no significant impact on the results for either the real or sham conditions for any of the cued-recall sessions. The sleep quality was not significantly different between real and sham stimulation before the first session, but significant difference was observed before the second session, where participants judged that they slept better before receiving sham stimulation (*p* = 0.008). A significant difference was detected in the arousal levels before the session between sham and real stimulation for the first day of memory cued-recall testing, where participants reported higher arousal levels in the real tDCS condition (*p* = 0.006), but not for the second cued-recall.

## Discussion

In the present study we investigate the effect of anodal tDCS over the left DLPFC on verbal-associative long-term memory performance. Interestingly, none of the stimulation conditions (before-during learning or before-during the recall phase) resulted in a modification of performance compared to the subjects during sham tDCS. Significant differences were observed between anodal and sham condition only in the sleep quality and arousal level in Group 2, however, without behavioral consequences.

Previous work has suggested that the excitatory-inhibitory balance in neuronal networks is disturbed during the learning of a new material (Song et al., 2000; Nabavi et al., 2014). In this period, when novel information is stored during the modification of excitatory synaptic strengths, anodal tDCS can have a beneficial effect and, consequently, augment the learning process. Indeed, data from numerous past experiments has implied that anodal tDCS can modify reaction time (for a review see Dedoncker et al., 2016b) or memory performance (for a recent review see: Hill et al., 2016) when administered in this critical period. However, the small effect sizes of previous studies, coupled with non-significant effects on several analyses, require cautious interpretation of these data. Moreover, since in the first experiment we fit the learning inside the last minutes of the stimulation protocol, homeostatic metaplastic effects could have driven the results toward a cancelation, as shown already when long-term potentiation-like brain stimulation protocols were applied prior to motor learning in humans (Jung and Ziemann, 2009).

With regard to the administration of anodal tDCS on the day after the verbal information encoding (experiment 2), we hypothesized that by decreasing the inhibitory rebalancing that is thought to take place after learning-induced increase in neuronal excitation (Froemke et al., 2007), a larger amount of semantic information would be recalled and memory performance would increase compared to sham stimulation. However, this was not the case and we were not able to replicate previous findings (Barron et al., 2016). Nevertheless, we can speculate that the timing of the stimulation in Group 2 might not have been ideal (e.g., the memory test should have occurred after the 20 min stimulation), as decreases in GABA levels were significantly lower after anodal tDCS applied at the primary motor cortex, but not during it, when compared to baseline measurements and to sham stimulation (Bachtiar et al., 2015). Another possible scenario is that the applied intensity might have been too low to generate an electric field strong enough to overcome the inhibitory effect. Nevertheless, the same intensity was used in previous research to successfully modulate GABA levels with anodal tDCS (Bachtiar et al., 2015; Barron et al., 2016).

Joyal and Fecteau (2016) reviewed studies that used tDCS in an attempt to modulate semantic processing. The data revealed a structured network correlated with this function, which included the inferior frontal gyrus, a region adjacent to the stimulated area but not directly targeted by it, a fact that can be related to the absence of significant behavioral outcomes. Moreover, we could speculate that the cathodal electrodes close to the lateral part of the frontal cortex may have driven this site to temporal inhibition. Additionally, findings on the cellular and network plasticity mechanisms that govern human learning and memory point to the fact that the search for a specific *locus* for the memory engram storage can be misleading, due to its possible widespread nature, i.e., the memory trace may be stored in connectivity patterns in different brain sites defined during encoding (Tonegawa et al., 2015). Therefore, targeting only one structure may be a limitation of each study with a design similar to the present one.

Furthermore, our target area might also not have been ideal. Indeed, multiple brain regions and not only the DLPFC are assumed to interact in order to coordinate verbal information processing, both for encoding and retrieval of declarative memories (Cabeza and Nyberg, 2000; Borst and Anderson, 2013; Pisoni et al., 2015). Besides, a fixed montage as chosen here can impact the study outcome. An individualized anatomical approach governed by neuronavigation to localize the DLPFC could optimize the results, in keeping with the known variability of brain anatomy as related to gender, aging, lateralization and pathological processes (Mylius et al., 2013). In addition, we employed electrode types that differed from the ones used in previous studies, that is, five smaller electrodes arranged in a Laplacian montage. Although, it was suggested by computational models and experimental studies that this sort of montage could effectively change cortical activity (Datta et al., 2009, 2012; Gbadeyan et al., 2016), the electric field induced by this montage is more focal and less deep compared to the one evoked by conventional pad electrodes.

Since we did not observe significant behavioral effects in the responses to tDCS, it would have been interesting to identify external and internal factors that might account for the negative results. Certainly, the variability in the cortical changes after tDCS that might result in the absence of group effects is a frequently discussed issue, particularly at the case of motor-evoked potentials responses after tDCS (e.g., Wiethoff et al., 2014). Many factors that could modulate responsiveness before, during or after tDCS have been identified (these being methodological-, investigator- and subject-related), however, until now, no consensus has been reached about the reasons underlying the between- and within-subject variability of tDCS effects. Although, it is difficult to compare directly, we believe that with regard to age, gender, educational level or sample size, this work did not differ from those published by previous research in tDCS and episodic memory.

So far, these studies in healthy participants have presented small effect sizes (Cohen's d of 0.04), with samples varying from 12 to 20 participants, with females outperforming male participants (Dedoncker et al., 2016a). Moreover, a meta-analysis that included only single-session protocols showed no significant effects of anodal tDCS on episodic memory (Dedoncker et al., 2016b). In another meta-analysis that included research with similar designs to the present work, only one study presented significant results, with a sample size of *n* = 16 (Horvath et al., 2015). Furthermore, in a recently published work (Emmerling et al., 2017), anodal tDCS was applied to the right DLPFC in order to manipulate cognitive control. The authors used the same experimental conditions in two independent experimental groups (with 18 and 16 participants receiving anodal stimulation), and surprisingly, after positive results in the first experiment, they failed to replicate their own previous findings in the second. They admitted that although this could have been related to insufficient power, the mechanisms underlying tDCS at the neuronal level are far from being understood.

Taken together, contrary to previously published results, we have found no evidence that single-session anodal tDCS over the left DLPFC with the parameters used in this work has a reliable effect on encoding and retrieval of verbal information in healthy adult subjects. These findings further highlight the importance of uncovering the methodological factors that might underlie inter-individual variability in response to tDCS, such as anatomical differences and electrode placement. Studies that combine behavioral outcomes with neurophysiological measures should systematically evaluate stimulation parameters and correlate them to effects in order to identify predictive factors. We also suggest that future studies should report not only the mean group data but also the individual performance data points. Moreover, to determine how far the negative results translate to a larger population, a higher number of participants is required.

## Ethics statement

This study was approved by the University Medical Center Göttingen Ethics Committee, under the title: Einfluss transkranieller Stromstimulation auf die kognitiven Funktionen. The project number is 12/04/2012, and it is registered under the IFS 1716.

## Author contributions

GL and AA designed the study. GL analyzed the data. PK and GL collected the data. GL, PK, WP, and AA wrote the paper.

### Conflict of interest statement

WP holds a patent on transcranial deep brain stimulation. He is on the scientific advisory board of Precisis AG. The other authors declare that the research was conducted in the absence of any commercial or financial relationships that could be construed as a potential conflict of interest.
